# Tissue-Welding Device: Considerable Advantages for Spleen Surgery Based on Histological and Cardiorespiratory Investigation

**DOI:** 10.1155/2022/9270966

**Published:** 2022-09-24

**Authors:** Linda Gatiņa, Inga Piginka-Vjaceslavova, Dace Bērziņa, Maksims Zolovs

**Affiliations:** ^1^Preclinical Institute, Faculty of Veterinary Medicine, Latvia University of Life Sciences and Technologies, Kr. Helmana Street 8, Jelgava LV-3004, Latvia; ^2^Institute of Food Safety, Animal Health and Environment ‘BIOR', Lejupes Street 3, Riga, Latvia; ^3^Department of Biosystematics, Institute of Life Sciences and Technology, Daugavpils University, Parades Street 1a, Daugavpils LV-5401, Latvia; ^4^Statistics Unit, Riga Stradins University, Balozu Street 14, Riga LV-1007, Latvia

## Abstract

During splenic surgery, it is important to control blood loss and the potential risk of cardiac arrhythmia. The best way to prevent complications from surgery is to use the appropriate surgical devices; however, there is no guideline for the use of specific surgical devices for spleen incision. Therefore, the aim of this research was to compare the interactions of various surgical devices with spleen tissue, their cardiorespiratory effects during incision, and subsequent spleen surgical wound healing. A total of 75 rabbits were included in the study. CO_2_ laser (*n* = 15), radiofrequency device (*n* = 15), electrocoagulator (*n* = 15), tissue-welding device (*n* = 15), and scalpel (*n* = 15) were used to make incisions in rabbits' spleens. Spleen biopsies of the incision area were taken from each animal at the day 0, 7, and 14 after surgery. Contactless thermography was performed during surgery. Suturing was not used after incision with the tissue-welding device, but incisions made by other surgical devices were sutured. The results showed that the width of spleen necrosis differed significantly between the various surgical devices used on spleen tissues. There was a positive, strong, and linear association between necrosis width and the tissue temperature of cutting edges. Significant increases in the heart rate were observed during spleen surgery performed with laser, scalpel, and radiofrequency devices. In conclusion, the tissue-welding device confers a significant advantage in spleen surgery, as there is neither a need for sutures nor a significant deviation in the heart rate.

## 1. Introduction

The spleen is a lymphatic organ that serves as the largest blood filter in the body, detects senescent and damaged cells, and plays a fundamental role in protecting the body from pathogens, via a combination of innate and adaptive immune responses [1–3]. The spleen also influences wound healing through a fibroblast-stimulating mechanism [3]. All functions of the spleen should be considered during splenic surgery. In human medicine, the negative effect of splenectomy has been studied for a long time and is very well documented [3, 4]. Sullivan et al. [4] investigated the immune response to the tridecavalent pneumococcal polysaccharide vaccine (prepared by Lederle Laboratories), which was significantly decreased in patients with anatomical or functional asplenia. Moreover, a high risk of sepsis development is present in asplenic patients [5].

By contrast, splenectomy and spleen-conserving resection in veterinary medicine are debated issues. There is no evidence of sepsis development in animals after splenectomy. Veterinary surgeons usually choose splenectomy because it is simple to perform and avoids intraoperative or postoperative bleeding.

Spleen injury could cause cardiac arrhythmia in a small animal [6]; however, it is not a common complication in humans [7]. There is much debate about the reasons for the occurrence of cardiac arrhythmia in patients with a spleen injury or following spleen removal. Patients with ruptured splenic masses have a higher incidence of cardiac arrhythmia, including ventricular tachycardia [8]. Pastarapatee and coauthors suggested that the sympathetic nervous system—enhanced catecholamine secretion and the release of myocardial depressant factor due to hypoxia or ischemia that can occur during surgery or the postoperative period—or an electrolyte imbalance may be involved in ventricular premature complex arrhythmia in dogs post splenectomy [8].

The use of appropriate surgical devices for spleen incision could reduce some of the complications of spleen surgery in veterinary and human medicine. There are no specific recommendations for splenic surgery in veterinary or human medicine; therefore, the study was focused on several surgical devices with different basic principles of tissue interaction. The electrocoagulator, tissue-welding device, and laser heat the tissue to the boiling point, which causes a rapid explosion of water in the cell and protein denaturation [9–11]. The thermal effect of an electrocoagulator on the tissue depends on the duration of application, the energy level, and the biological properties of the tissues [1, 2, 4–6, 8–10, 12–28]. A laser causes thermal damage, necrosis, and carbonization, not only in the damaged bone tissue but also in the surrounding soft tissues [1, 5, 6, 8, 9, 12–22]. There is minimal information on the tissue-welding device and the healing process following its use, but the hypothesized working principle of tissue-welding devices is based on the denaturation of albumin, collagen, and elastin in the blood vessel walls [29]. The radiofrequency device increases the temperature of internal structures of the cells, intracellular fluid is lost and protein denaturation occurs [12, 28]. The narrow range of radiofrequency waves ensures reduced heating of the surrounding tissues and can almost completely prevent thermal damage [10, 12, 30]. Each surgical device has a different effect on tissue; therefore, the aim of this research was to compare the interactions of various surgical devices with spleen tissue as well as their cardiorespiratory effects during incision and subsequent spleen surgical wound healing.

## 2. Materials and Methods

### 2.1. Experimental Design

The study was carried out at the Faculty of Veterinary Medicine, Latvia University of Life Sciences and Technologies from 2015 to 2018. A total of 75 male Czech spot breed rabbits, 16–20 months of age (average 17 ± 1.29), average weight 3.0 ± 0.23 kg, with body condition score of 3 (scoring range from 1.0 to 5.0, Size-O-Meter) [27], were randomly divided into five experimental groups. A tissue-welding device, CO_2_ laser, electrocoagulator, radiofrequency device, and conventional scalpel were used to make the incision in the respective experimental groups. At the beginning of the study, all rabbits were clinically healthy, the body temperature, heart and breathing rate was at physiological norms, and the auscultation of heart and lungs showed no abnormalities. The mouth mucosa was pink and moist. There was no discharge observed from rabbits' eyes, ears, or nose. The superficial lymph nodes (mandibular and popliteal lymph nodes) were palpated and were normal. The rabbits were kept in individual cages, with a floor space of 0.5 m^2^ and a height of 50 cm. Every animal had a place to hide in a pipe that imitated a cave [19]. The environment was maintained at 18–20°C and 55%–60% relative humidity, and access to food and water were in line with animal welfare standards. To eliminate stress, the room where all rabbits were kept was closed at all times to exclude noise and environmental changes. One person performed all the animal maintenance, such as feeding, clean water refiling, and cage cleaning.

The animals received an intramuscular injection of 10% ketamine (35 mg/kg) and medetomidine hydrochloride (0.5 mg/kg). As soon as the absence of palpebral reflex was recorded, the animals were connected to an inhalation anesthesia device (Eickemeyer IsoFlo), and further anesthesia was provided using IsoFlo 100% (0.25–2.0%). To determine the heart rate and read the electrocardiogram and blood oxygen levels for all 75 rabbits during surgery, the monitoring sensors “BM3-vet” was used. Analgesic medications (meloxicam 0.2 mg/kg) with a 24-hour effect were injected intramuscularly at the beginning of the operation. After surgery, antibiotics (enrofloxacin 5 mg/kg) were injected subcutaneously. For the next four days, rabbits received meloxicam 1 mg/kg per os every 24 hours. Standard environmental conditions were maintained in the surgery room: the temperature was 20–22°C and the relative humidity was 40%–45% [31].

The surgical approach using a different surgical device in each group was applied as follows: for three animal groups, an incision was made at the dorsal end of the spleen diaphragmatic surface by (a) a CO_2_ laser “Aesculight” (*n* = 15) 2.8 mm in depth, (b) a radiofrequency apparatus “Curis” (*n* = 15) 1.3 mm in depth, and (c) a conventional scalpel (*n* = 15) 1.8 mm in depth. The length of the incision made with all of these three devices was approximately 1–1.5 cm, based on the recommendation of Food and Veterinary Service experts, who evaluate the experimental methods involving animals. For the other two animal groups, 4 mm of the spleen's dorsal end was removed using a scissor-like hand tool called an electrocoagulator “BOVA ARC 250” (*n* = 15) and a tissue-welding device “EK-300M1 MAC” (*n* = 15). The wounds were sutured after incision with a CO_2_ laser “Aesculight,” a radiofrequency apparatus “Curis,” an electrocoagulator “BOVA ARC 250,” and a scalpel, using simple, interrupted sutures with monofil Monocryl 2-0 suture material, but the incision made with the tissue-welding device was not sutured. The average rabbit spleen length was 5–6 cm and the width 0.7–0.77 cm; therefore, incision in the spleen, which was approximately 0.4 cm long, was comparable to partial splenectomy [20].

The settings of these devices were adjusted according to the manufacturers' recommendations and the manuals provided. The laser was set in a super pulse energy emission mode in continuous exposure mode, with a laser focus of 0.4 mm^2^ and a power output of 7 Watts (W) selected. The electrocoagulator was set in “cut mode” with bipolar output and a maximum power of 120 W. The radiofrequency device was set in standard mode “cut 1” with a maximum of 7 W and the monopolar hand tool. The tissue-welding device was set in cutting mode and 70°C maximum heat.

To evaluate the thermal effects of these devices on the spleen, contactless thermography by the “Flir-i3” thermograph was performed at an acceptable distance of 5 cm from the cut site at a 45° angle using a method adapted from Ref. [16]. Thermographic imaging was performed during the use of various surgery devices and the scalpel. All images were obtained by the same person to ensure consistency. Thermographic changes on the obtained images and the focal point of thermal measurements were analyzed using the “FLIR Quic Report 1.2 SP2” program.

### 2.2. Histological Examination

Spleen biopsy samples, 0.5–0.75 cm^3^, were taken three times: immediately after the incision-day 0 (*n* = 5), day 7 (*n* = 5), and day 14 (*n* = 5) after the surgery. The biopsy samples were taken by cutting 4 mm of the dorsal end of the spleen on day 0, including the cut surface and surrounding tissue. The biopsy taken on days 7 and 14 included the scar tissue and surrounding tissue.

Biopsy samples were fixed in 10% formalin. After 48 hours of fixation, the samples were transferred to cassettes and placed in the “Tissue strainer MEDITE” tissue processor. After processing, the samples were sliced using a “Leica RM2255” microtome and mounted on slides. Slides were stained with hematoxylin and eosin.

Tissue histological examination was performed using a “Leica DM500” microscope, and measurements of necrotic tissue and granulation and changes in cellular details in the tissue capsule and wound edge tissue were performed using the “LAS V4.7” program.

### 2.3. Statistical Data Analysis

The assumption of normality of the data distribution was assessed by the Shapiro–Wilk test and inspection of the normal Q-Q plots. The assumption of homogeneity of variances was tested by Levene's test. The presence of outliers was inspected according to Hoaglin [13]. The Kruskal–Wallis H test was performed to determine whether there was a significant difference between the five independent groups. Post hoc analysis was conducted by applying the Dunn test. The Mann–Whitney *U* test was conducted to determine whether there was a significant difference between the two independent groups. A dependent samples *t*-test was conducted to determine whether a difference existed between the means of two related groups for a continuous dependent variable. Spearman's correlation test was conducted to determine the strength and direction of the linear relationship between necrosis width and granulation tissue width, as well as necrosis width and temperature regimes of surgical devices application.

## 3. Results

**Figure 1:**
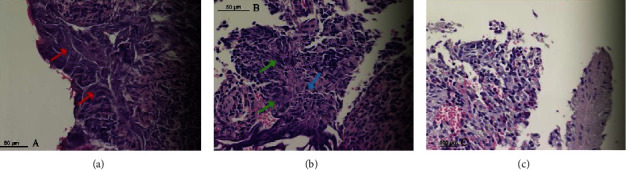
Different cellular changes following the use of various cutting methods. (a).Cut made with a tissue-welding device, obscured and disorganized cells (red arrow). (b).Cut made with a laser, starched nucleus (green arrow) and karyolysis (blue arrow). (c).Cut made with a scalpel with no cellular changes.

**Figure 2:**
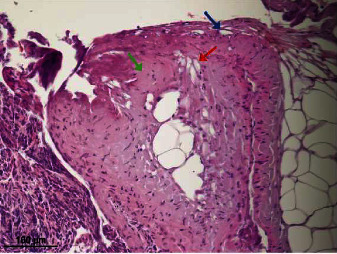
Vacuolation (red arrow), cleft formation (blue arrow), and hyalinization of collagen (green arrow) in spleen capsule following the use of a tissue-welding device.

**Figure 3:**
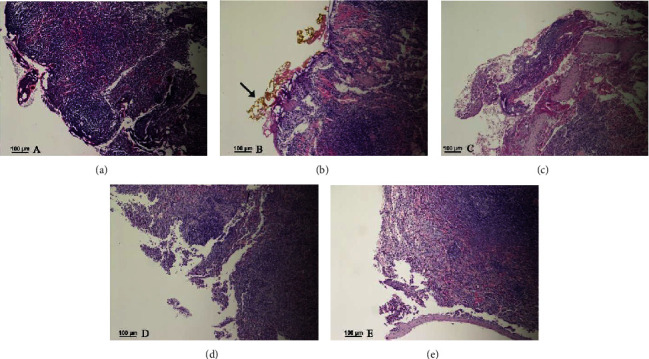
Differences in wound edges following the use of various cutting methods. (a).Uneven wound edge of cut made with an electrocoagulator. (b).Smooth straight wound edge of cut made with a laser, yellow to black pigment accumulation (carbonization) near the wound edge (arrow). (c).Uneven wound edge of cut made with a tissue-welding device. (d).Uneven, torn wound edge of cut made with the radiofrequency device. (e).Smooth, slightly uneven wound edge of cut made with a scalpel.

The data on histological changes in tissue were compared on day 0 after using various surgical devices, and the results are summarized in Table 1. Significant changes in cellular details were seen in all tissue samples where devices with an excessive heat effect were used (Tables 1 and 2). Obscured and disorganized necrotic cells with stretched nuclei, karyolysis, and vacuolization in nuclei were observed in these samples (Figure 1). Vacuolization was present in the capsule after tissue welding and laser use (Figure 2). One cutting method with a low heat effect (radiofrequency device) and another without a heat effect (scalpel) on the tissues did not show necrotic changes in wound edges. Smooth wound edges with a capsule attached to the parenchyma were only observed after laser use, but after the use of other devices, uneven wound edges with complete or incomplete capsule separation of the parenchyma were detected (Figure 3). However, there were certain similar changes in some of the cutting methods with and without a heat effect on the tissue. Cleft formation and cell separation were observed in all of the biopsy samples (Figure 2).

On day 0, brown to yellow pigment accumulation was observed on the edges of the wound from tissue carbonization by laser cutting only. On days 7 and 14, carbonization pigments were phagocytized by multinucleated giant cells and were observed in the vicinity of wounds caused by the laser and also by the electrocoagulator.

Complications like abdominal bleeding during surgery or postoperatively were not observed. In histological samples taken on day 0, mild-to-moderate hemorrhage near the wound edge in each group of samples was observed. Hemorrhage was seen in all samples where cuts were made with scalpels. The laser caused the least hemorrhage, which only occurred in two samples. In cuts made with the tissue-welding device, radiofrequency device, and electrocoagulator, hemorrhage was visible in three to four samples.

**Figure 4:**
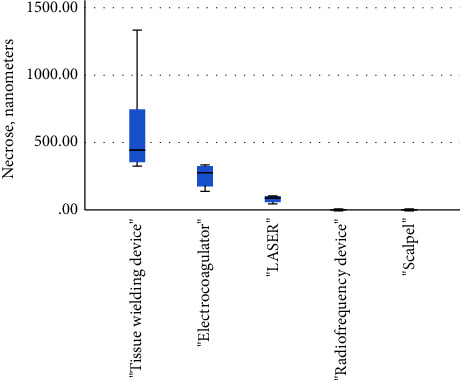
The width of spleen necrosis after the use of various surgical devices on day 0.

The width of spleen necrosis differed significantly between surgical devices x^2^(2) = 11.06 *p*=0.003, *ε*^2^ = 0.83 (high strength). The large effect size indicated that the difference had practical meaning. Post hoc analysis revealed that the width of spleen necrosis differed significantly between the tissue-welding device and the laser, as well as between the laser and the electrocoagulator. Detailed results are summarized in Table 3 and presented in Figure 4.

The Mann–Whitney *U* test showed that there were no significant differences in the width of the granulation tissue with and without ligation after incision. The Spearman's correlation test showed that there was a strong, positive, and linear association between necrosis width and the temperature regimes used in surgical device application *r*_s_ = 0.523, *n* = 25, and  *p*=0.007, indicating that the temperature increased, necrosis width also tended to increase. The laser heated the tissue the most—up to 187.03°C, but the radiofrequency device heated the tissue the least—up to 47.05°C of all the electrosurgical devices. The results showed that there was no significant difference in granulation tissue formation in any of the biopsy samples taken from all of the cutting methods on days 7 and 14 after the surgery (Table 2).

The results of respiratory and heart rate comparison between incisions made in the skin and in the spleen are summarized in Tables 3 and 4. The heart rate differed significantly between use of the radiofrequency device (*p*=0.016) and the scalpel (*p*=0.001). The effect size (*d*) shows that the difference obtained had practical meaning. All three devices had high effect sizes. Additionally, breath fervency was evaluated according to the various surgical devices used.  Table 4 shows no significant effect of various surgical devices on the respiratory rate.

## 4. Discussion

The choice of a surgical device can have a variable effect on incision edges. The study included several cutting methods with different hand tools and cutting depth. The cuts in the spleen with laser, radiofrequency device, and scalpel were done with a single cut with the handpiece to avoid from additional physical and thermal trauma of using the cutting methods several times. The depth of cut depends on specifics of each cutting method and the depth that the cutting method can reach in one use. Devices that have a high thermal effect on tissue cause necrosis with cellular karyorrhexis. A radiofrequency device did show tissue necrosis with the use of this device, possibly because radio waves have a direct effect on the extracellular matrix and separate the tissue between the cells. Additionally, these electrical devices provide hemostasis: the laser, electrocoagulator, and radiofrequency device by coagulating proteins, and the tissue-welding device by coagulating collagen and sealing blood vessels [12,29]. In recent years, limited information and scant research have been published on the provision of hemostasis during spleen surgery. In 2006, Itamoto et al. published a case report [14], in which they concluded that radiofrequency could be used to perform a partial splenectomy with no bleeding and is valid for use in spleen surgery. Signs of bleeding were not observed in the abdomens of animals during surgery or in later postmortem examinations. Histologically, mild-to-moderate acute hemorrhage was observed on the surgical wound edges in the spleen on day 0 and chronic hemorrhage with hemosiderin accumulation on days 7 and 14. The presence of hemorrhages around the incision edges was anticipated due to the vascular anatomical structure [32].

It is necessary to mention that the spleen contains many small blood vessels, and the walls of arteries and veins contain collagen [26]. In fact, collagen coagulation was observed in the capsule but was not on blood vessel walls in the incision made by the tissue-welding device. However, there was no intra-abdominal bleeding from the spleen incision made using a tissue-wielding device without sutures. Presumably, the tissue-welding device activated fibrin coagulation in addition to collagen coagulation. The effect of the tissue-welding device on the spleen tissue was adequate to protect the incision from bleeding without suturing. This is a very important feature, as it allows for a rapid and safe surgical approach. It is known, that in human medicine, partial splenectomy is often used to remove nonparasitic hydatid splenic cysts [17]. The surgical approach could be improved using the tissue-welding device, and surgery on the spleen can be performed without stitches. Of course, the use of these devices in human medicine for partial splenectomy should be approved.

In 2018, Kashyap [15] describes laser incision as bloodless during surgery and the postoperative period, in contrast to scalpel incision. Likewise, in the study, the use of a laser produced better results regarding hemorrhage immediately after incision, where only two out of five animals showed acute hemorrhage at the incision edges.

The results showed that the use of an electrocoagulator can prevent bleeding during surgery, but research by Watts in 2018 [33] detected only one case of 3744 animals (cats and dogs) with postoperative bleeding after castration with a bipolar electrocoagulator.

Although electrosurgical devices can prevent bleeding during surgery, these devices lead to the necrosis at the wound's edges. The study showed significant expansion of necrosis at the incision edges, with increasing temperature effects of the surgical devices on the tissue. The width of necrosis depends not only on the temperature range from these devices at the incised edges but also on the distribution of the temperature effect in the tissue. The narrowest necrosis of wound edges was detected after the use of the laser despite the highest temperature effect of the laser on the wound edges. It is possible that the laser affects a wider area of tissue due to the laser beam width, resulting in a broader effect on the tissue compared with that of other surgical devices. The width of splenic necrosis depends significantly on the surgical devices used, and this difference has practical meaningful. Necrosis of the spleen incision caused by the tissue-welding device, electrocoagulator, and laser was significantly more extensive than that caused by the scalpel and radiofrequency device. In 2021, Carqueville and Chesnut [34] also concluded in their study that the use of laser and electrocautery caused the wider area of necrosis in skin than did a conventional scalpel.

The appearance of necrosis caused by various surgical devices is variable and depends on the impact of the surgical devices on the tissues and direct contact with the tissue. The laser, electrocoagulator, and tissue-welding device showed specific cellular changes: obscured and disorganized cellular details and stretched nuclei with karyolysis. In cuts made with a laser and electrocoagulator, carbonization was observed as a yellow to brown pigment on the wound edge, which appeared immediately after laser incision and later after use of the electrocoagulator. The laser (187.3°C) and the electrocoagulator (73.25°C) reached the highest temperatures during incision. In 2018, Kashyap et al. [15] also described the connection between temperature and the amplitude of tissue damage by comparing the effects of a laser and scalpel on tissue during oral biopsy sampling and also observed carbonized tissue in 85% of the laser biopsy samples. This carbonization irritates the tissue; therefore, histiocytes combine and fuse together to form multinucleated foreign body giant cells in the tissue and attempt to phagocytize carbonized tissue [25].

Chu et al. [35] described the importance of the absence of contact force and carbonization during cardiac surgery with radiofrequency catheters. Rebling et al. [21] also asserted that radiofrequency devices did not cause carbonization during cardiac surgery. The study results show very similar wound edges appearance in surgery with a radiofrequency device and scalpel. Neither device uses heat to achieve its cutting function; therefore, the edges of the wound showed cleft formation and cell separation without cellular changes or tissue carbonization.

Despite the irritation of carbonatized tissues and other thermal effects, the study results show that all incisions made in the spleen by various surgical devices with and without thermal effects are similarly healed on day 7 after surgery, with proliferation of granulation tissue.

Histological evaluation on day 0 showed a difference in the edge of the wound. Smooth wound edges were detected using a laser for spleen tissue incision, this could be a result of the thin laser beam, which made a cut as thin as a sharp object. Similar changes were described in 2011 by Saeed and Mahmood [22], where a cut was made in the rabbit tongue with laser. The study shows that other surgical devices, such as the scalpel, radiofrequency device, electrocoagulator, and tissue-welding device, produced an incision with uneven or slightly uneven wound edges. Despite differences in the edge of the incision wound, the study results showed that all incisions made by various surgical devices were similarly healed on day 7 after surgery with granulation tissue proliferation. There was no significant difference in healing and granulation tissue formation in incision wounds with and without ligation. Therefore, the tissue-welding device can be used safely in spleen surgeries without ligations. Worthy of note, in 2009, Stronceks et al. [25] detailed that sutures such as poliglecaprone (Monocryl) are slowly absorbed within 100 days or more; therefore, tissues reactions around absorbable sutures can be characterized by an increased inflammatory response, fibroblasts depositing a collagen matrix around the suture, and macrophages fusing to form multinucleated giant cells in an attempt to degrade the suture material. According to the study, it is possible to avoid these chronic inflammatory reactions by using a tissue-welding device for spleen surgery without stitches. The results did not show significant differences in the width of granulation tissue between day 7 and day 14 after surgery in all incisions made by various surgical devices. This means that the incision wound was fully healed on day 7 after surgery in all animals including cases where surgery was performed without stitches.

The application of these surgical devices could have not only a local effect at the site of the spleen incision but also a general effect in all organisms, and such effects could be unwelcomed. In veterinary medicine, one of the most commonly encountered complications after a splenectomy is ventricular cardiac arrhythmias [18,24]. None of the animals of this study experienced cardiac arrhythmia during surgery. However, a significant increase in the heart rate was observed when cuts were made in the spleen compared with the heart rate during the skin incisions made by laser, scalpel, and radiofrequency devices. Most available studies conclude that the reason for ventricular arrhythmias during and after splenectomy includes cardiosympathetic activity through stimulation of the splenic nerve and myocardial hypoxia due to lower blood loss [24]. The patients of this study did not lose a lot of blood, and the splenic nerve was not damaged during of spleen incision as it would be in the case of splenectomy. This could be the reason for the absence of ventricular arrhythmia, but it appears that irritation of the spleen during surgery was sufficient to cause elevation of the heart rate. In a 2020 study including over 200 dogs, Sirochman et al. in 2020 [23] concluded that intraoperative ventricular arrhythmias were significantly associated with death prior to discharge after splenectomy when a bipolar vessel sealing device was used. The bipolar vessel sealing device (electrocoagulator) and tissue-welding device in this study had no significant effect on the heart rate during spleen surgery.

## 5. Conclusions

In conclusion the tissue-welding device confers a significant advantage in spleen surgery, as there is neither a need for sutures nor a significant deviation in the heart rate.

## Figures and Tables

**Table 1:** Tissue changes caused by various surgical devices immediately after incision.

Histological Observation	Surgical devices				
	Tissue-welding device	Laser	Electrocoagulator	Radiofrequency device	Scalpel
Wound edge (Figure 1)	Uneven	Smooth straight with yellow to black pigment accumulation (carbonization)	Uneven	Uneven, torn	Smooth, slightly uneven

Cellular changes (Figure 2)	Obscured and disorganized, stretched nuclei, karyolysis	Obscured and disorganized, stretched nuclei, karyolysis	Obscured and disorganized, stretched nuclei, karyolysis	No visible changes	No visible changes

Tissue changes	Cleft formation and cell separation	Deep clefts, vacuolation, and cell separation	Cleft formations filled with red blood cells, vacuolation, and cell separation	Cleft formation, cell separation, and vacuolation	Cleft formation, cell separation

Capsule	Vacuolation, hyalinization of collagen (Figure 3)	Vacuolation and cell separation	Total separations of the capsule from the parenchyma	Total separations of the capsule from the parenchyma	Total separations of the capsule from the parenchyma

**Table 2:** Difference in rabbit heart rate between skin and spleen incision (beats per minute).

Surgical devices	Skin incision		Spleen incision		*p*	*d*
	M	SD	M	SD		
Tissue-welding device	150	15.02	155	20.05	0.476	0.189
Laser	177	15.01	186	11.06	0.002	1.030
Electrocoagulator	145	30.07	151	39.09	0.276	0.293
Radiofrequency device	140	19.04	155	23.08	0.016	0.708
Scalpel	150	25.01	167	21.05	0.001	1.540

M-mean, SD-standard deviation, *d-*effect size.

**Table 3:** Thermoeffect of various surgical devices on the tissue on experimental day 0 and granulation tissue on day 7 and day 14.

Type of surgical device	Suture used	Median tissue temperature °C (IQR) Day 0	Median tissue necrosis in *μ*m (IQR) Day 0	Median of granulation tissue in *μ*m (IQR)		*p* Value
				Day 7	Day 14	
TWD	No	57.01 (49.1–66.4)	443 (354–758)	-	-	-
Laser	Yes	187.03 (168.3–234.1)	86.08 (66.6–94)	1123 (562–1498)	497 (378–531)	0.188
EC	Yes	73.25 (69.23–79.28)	281 (171–335)	2044 (1175–2811)	1910 (1403–1935)	0.813
RD	Yes	47.5 (45.2–50.1)	0	361 (298–517)	392 (266–477)	1
Scalpel	Yes	35 (34.6–35.4)	0	974 (683–1575)	357 (254–435)	0.125

IQR-interquartile range presented as Q1–Q3, TWD – Tissue-welding device, EC-Electrocoagulator, RD-Radiofrequency device.

**Table 4:** Difference in rabbit respiratory rate between skin and spleen incision(breaths per minute).

Surgical devices	Skin incision		Spleen incision		*p*	*d*
	M	SD	M	SD		
Tissue-welding device	40.08	7.01	39.03	02.08	0.437	0.223
Laser	58.04	31.03	51.04	22.08	0.242	0.316
Electrocoagulator	35.03	6.62	35.09	05.07	0.612	0.134
Radiofrequency device	25.0	13.02	29.01	15.0	0.204	0.357
Scalpel	43.05	17.05	42.09	14.02	0.811	0.062

SD-standard deviation, *d-*effect size, M-mean.

## Data Availability

Data used to support the findings of this study are available from the corresponding author upon request.
